# Comprehensive Evaluation of Frailty and Sarcopenia Markers to Predict Survival in Glioblastoma Patients

**DOI:** 10.1002/jcsm.13809

**Published:** 2025-04-15

**Authors:** Chao Yang, Chao Ma, Cheng‐Shi Xu, Si‐Rui Li, Chen Li, Ze‐Fen Wang, Zhi‐Qiang Li

**Affiliations:** ^1^ Department of Neurosurgery Zhongnan Hospital of Wuhan University Wuhan China; ^2^ Department of Radiology Zhongnan Hospital of Wuhan University Wuhan China; ^3^ Department of Physiology Wuhan University School of Basic Medical Sciences Wuhan China; ^4^ Department of Clinical Nutrition Zhongnan Hospital of Wuhan University Wuhan China

**Keywords:** glioblastoma, mean corpuscular volume, prognosis, prognostic nutritional index, sarcopenia, temporal muscle thickness

## Abstract

**Background:**

Glioblastoma (GBM) is the most common primary malignant brain tumour in adults. Patients with GBM are particularly susceptible to moderate‐to‐high frail. Frailty status has been associated with the outcome of many types of cancer, including GBM, although there is still little consensus regarding the specific criteria for assessing frailty status. This study aimed to determine the predictive significance of the modified frailty score (mFS) in GBM patients using haematological and sarcopenia indicators.

**Methods:**

Between January 2016 and September 2022, we enrolled 309 adult GBM patients. Data on demographics, haematological examination, and temporal muscle thickness (TMT) were collected and assessed. The prognostic relevance of the frailty parameters was established using Kaplan–Meier and Cox proportional model. The scoring systems were created by integrating these indicators. Variables with independent prognostic values were used to construct the nomograms. Nomogram accuracy was evaluated using the calibration curve, Harrell's concordance index (C‐index), and time‐dependent receiver operating characteristic curves. Clinical practicality was assessed using decision curve analysis.

**Results:**

The baseline characteristics of the 309 participants revealed a median age of 59 years (interquartile range 52–66) with a predominance of male patients (58.58%). TMT (hazard ratio [HR] = 3.787, 95% confidence interval [CI] 2.576–5.566, *p* < 0.001), prognostic nutritional index (HR = 1.722, 95% CI 1.098–2.703, *p* = 0.018), and mean corpuscular volume (HR = 1.958, 95% CI 1.111–3.451, *p* = 0.020) were identified as independent prognostic markers. The constructed mFS, obtained by integrating these three indices, exhibited independent prognostic significance (HR = 2.461, 95% CI 1.751–3.457, *p* < 0.001). The patients in the low‐risk group had a median overall survival (OS) of 13.9 months, while the patients in the high risk had a median OS of 5.8 months. Importantly, the mFS demonstrated significant independent prognostic value in the subgroup aged > 65 (HR = 1.822, 95% CI 1.011–3.284, *p* = 0.046). The nomogram, which included the mFS, demonstrated high accuracy, with a c‐index of 0.781. The nomogram bootstrapped calibration plot also performed well compared to the ideal model. Nomograms showed promising discriminative potential, with time‐dependent areas under the curves of 0.945, 0.835, and 0.820 for 0.5‐, 1‐, and 2‐year overall survival prediction, respectively.

**Conclusions:**

Preoperative mFS is a comprehensive frailty marker for predicting survival outcomes in patients with GBM. A dynamic nomogram incorporating the mFS may facilitate preoperative survival evaluation. Early and appropriate multimodal interventions, including nutritional support, rehabilitation, and psychological care, may help in the neurosurgical care of patients with GBM or other brain tumours.

## 1 Introduction

The incidence of primary malignant brain tumours stands at roughly 7 cases per 100 000 individuals, with glioblastomas (GBMs) accounting for nearly 49% of these cases [1]. Despite substantial advancements in GBM treatment in recent years, the prognosis remains poor, with a median overall survival (OS) of approximately 15 months [2]. Even among patients receiving the same therapy, a considerable variation exists in their 5‐year survival rates, ranging from 5.5 to 28.5%. Thus, identifying reliable prognostic indicators is crucial to aid in patient classification and treatment plan development in clinical practice. Current prognostic indicators for GBM widely used include Karnofsky performance scale (KPS) score, tumour resection extent, and O6‐methylguanine‐DNA methyltransferase (MGMT) promoter methylation status [3]. However, the precision of these markers is limited, and the acquisition of molecular markers remains restricted to the postoperative period. Therefore, developing more practical and accurate prognostic indicators for patients with GBM is imperative.

Frailty is a well‐known but overlooked aspect of surgical oncology. It occurs in approximately 51% of patients and is caused by a combination of factors, including insufficient calories, reduced activity, systemic inflammation, and metabolic changes induced by the tumour and its treatment [4]. Patients with cancer, especially older patients with comorbidities, are particularly susceptible to moderate‐to‐high frailty status during radiotherapy, chemotherapy, immunotherapy, surgery, and drug treatment. However, timely and effective frailty screening, assessment, diagnosis, and intervention are lacking. This deficiency leads to inadequate, inappropriate, or delayed nutrition therapy, which exacerbates complications, prolongs hospital stays, increases readmission rates and mortality, and lowers the quality of life. Additionally, it adds to the burden on healthcare systems, families, and the society as a whole. Energy consumption, weight reduction, body composition, fluid build‐up, and functional status have been used to evaluate frailty status [5]. The Nutritional Risk Screening 2002, launched by the European Society for Clinical Nutrition and Metabolism in 2003, is the most widely used tool to assess the nutritional risk of all patients who are hospitalised [6]. However, it often presents limitations in evaluating the nutritional risk of patients with brain tumours. For example, bedridden patients may struggle to measure their weight, and conditions such as oedema or ascites can impact the accuracy of the measurement of weight, BMI, skinfold thickness, and muscle circumference, as well as body composition analyses, including dual‐energy X‐ray absorptiometry and bioelectrical impedance analysis. Additionally, this scale may not be applicable to patients with impaired consciousness. Hence, exploring more objective, effective, and practical frailty assessment indicators for patients with brain tumours is obligatory.

Serum albumin [7], total lymphocyte count [8], and related markers such as the albumin‐to‐globulin ratio (AGR), and prognostic nutritional index (PNI) are among the serum biomarkers that have recently been studied. The predictive value of AGR has been reported for many cancers, including GBM [9]. Similarly, several studies have confirmed that PNI can be an effective prognostic biomarker in several types of malignancies, including gastrointestinal cancer [10], and GBM [11]. In addition to haematological markers, sarcopenia is an objectively measured parameter associated with poor prognosis and frailty in several extracranial malignancies [12]. Computed tomography (CT) is used to measure the skeletal muscle cross‐sectional area at the third lumbar vertebra to assess sarcopenia. However, radiological images of the abdomen are usually not readily available during routine neurosurgery. Recent research has shown a strong relationship between the cross‐sectional areas of the lumbar skeletal muscles and temporal muscle thickness (TMT) measured on diagnostic brain magnetic resonance imaging (MRI) [13]. This suggests that skeletal muscle mass can be estimated using the lumbar and craniofacial muscles. Researchers have demonstrated that TMT can be used as an independent prognostic parameter for patients with primary central nervous system (CNS) lymphoma [14], brain metastasis [15], and GBM [16, 17]. Moreover, according to a retrospective study by Yan et al., which included 261 patients with glioma, a thicker TMT was associated with longer OS in gliomas of various grades and isocitrate dehydrogenase (IDH) subtypes [18]. However, the prognostic value of TMT in GBM remains controversial. Muglia et al. reported that patients with higher TMT did not show a statistically significant increase in OS [19]. Another study revealed that TMT cannot predict postoperative survival outcomes in patients with primary GBM [20]. A close correlation exists between tumour progression, frailty status, and cachexia. Growth differentiation factor 15 (GDF15), a member of the transforming growth factor superfamily, has been extensively researched in terms of its relationship with obesity, cachexia, and vascular diseases [21]. GDF15 could enhance the malignancy of gliomas [22], and elevated levels of GDF15 in cerebrospinal fluid are conspicuously associated with poorer prognoses in GBM [23]. Considering that diverse types of frailty indicators might influence the prognosis of cancer patients via distinct mechanisms, a scoring system founded on various types of frailty indicators can offer a more accurate and comprehensive assessment of survival. Importantly, both haematological markers and TMT based on brain MRI are routine indicators available in neurosurgical clinical practice. Furthermore, at present, there is a relative dearth of scoring systems based on multi‐dimensional frailty indicators for GBM patients.

This study aimed to assess the predictive importance of frailty status in patients with GBM by integrating demographic, haematological, and sarcopenia indicators. The survival probability of each patient was predicted using nomogram models that combined multiple independent prognostic factors.

## 2 Materials and Methods

### 2.1 Inclusion and Exclusion Criteria for the Study Cohort

This study included 309 adult patients with GBM treated at Zhongnan Hospital of Wuhan University between January 2016 and September 2022. Inclusion criteria included the following: (1) age ≥18 years at diagnosis; (2) all diagnoses confirmed pathologically by two pathologists independently, using the 2021 World Health Organization classification of CNS tumours; (3) pre‐operative peripheral blood test data and associated clinical information were accessible; (4) the brain MRI, using post‐contrast three‐dimensional (3D) magnetisation‐prepared rapid acquisition with gradient echo (MPRAGE), was performed at our institution on a 3‐T scanner (Magnetom Verio, Siemens Healthineers) no later than 3 days before surgery.

**FIGURE 1:**
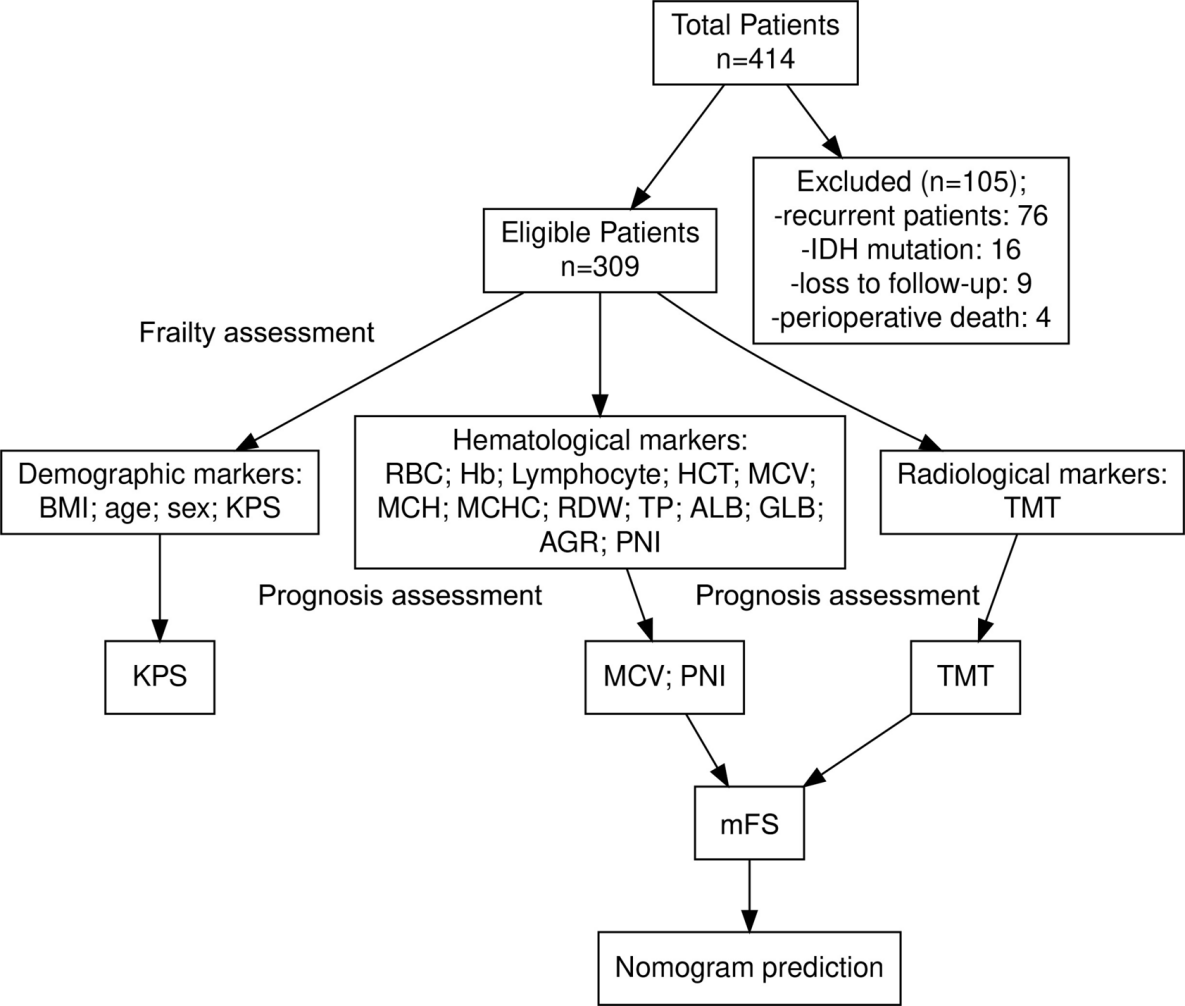
Flow diagram of the present study.

The exclusion criteria were as follows: (1) receiving chemotherapy or radiation treatment (including corticosteroids) prior to surgery; (2) patients who died perioperatively; (3) a history of other malignancies; (4) recurrent GBM; (5) existing diabetes, hyperthyroidism, liver cirrhosis, and other metabolic disorders; and (6) any treatment that altered the thickness of the temporal muscle (such as prior craniotomy or radiotherapy). The study adhered to the principles outlined in the Declaration of Helsinki. The last follow‐up was conducted on 30 December 2023. Additionally, we used the STROBE checklist for reporting this study (Supplementary File S1). Figure 1 shows a flow diagram of the study.

### 2.2 Data Collection

Demographic and clinical information including BMI, age, sex, KPS, tumour volume, tumour resection extent (gross total resection [GTR]: no measurable radiographic lesions; subtotal resection [STR]: have measurable radiographic lesions), completion of postoperative chemotherapy and radiotherapy, and MGMT promoter methylation status were obtained. Additional information regarding the patient's peripheral blood test results was collected before surgery. These included red blood cell (RBC) and lymphocyte count; haemoglobin (Hb) level; haematocrit (HCT); mean corpuscular volume (MCV); mean corpuscular haemoglobin (MCH); mean corpuscular haemoglobin concentration (MCHC); RBC distribution width (RDW); and total protein (TP), albumin (ALB), and globulin (GLB) levels. The PNI (albumin concentration [g/L] + 5 × lymphocyte count [10^9^/L]) and AGR were computed using these parameters. OS was calculated from the time of surgery to death or the last follow‐up. Follow‐up assessments were performed on an outpatient basis or via telephone.

### 2.3 TMT Measurement

**FIGURE 2:**
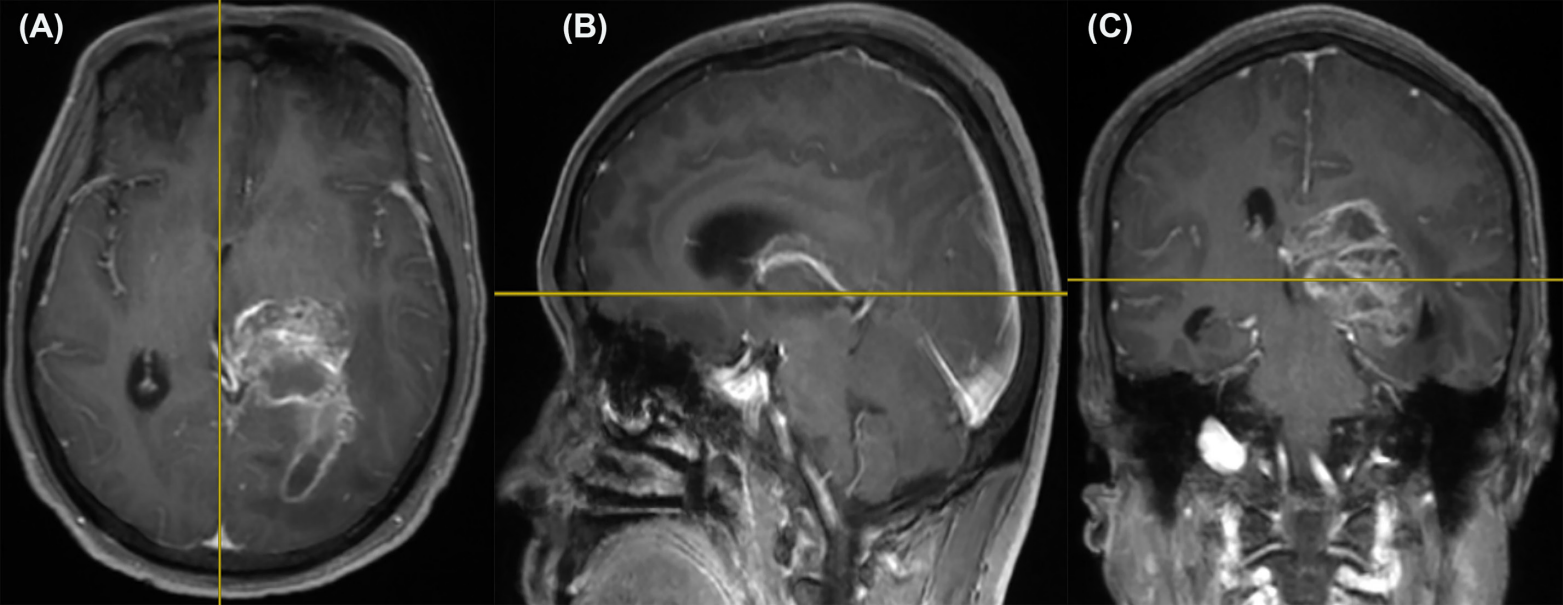
Typical pictures illustrating TMT measurement. Before surgery, axial (A), sagittal (B), and coronal (C) views were applied to post‐contrast 3D MPRAGE images obtained on a 3‐T scanner within 72 h. Using the orbital roof and the Sylvian fissure as reference points, TMT was measured on an axial plane that ran parallel to the anterior–posterior commissure line. TMT, temporal muscle thickness.

TMT was measured using presurgical brain MRI scan, imported into the RadiAnt Dicom Viewer program (version 5.5; Medixant, Poland). Subsequently, two radiologists employed multiparametric reformatting to refine the 3D‐MPRAGE sequences across three orthogonal planes (axial, coronal, and sagittal) through consensus. The axial plane was positioned parallel to the anteroposterior commissure line. Employing the orbital roof and Sylvian fissure as anatomical indicators, the thickness of the temporal muscles was measured bilaterally, perpendicular to the long axis of the muscle, in accordance with a previously published method for TMT assessment [16] (Figure 2). The mean TMT was determined by independently measuring it both on the left and right sides of each patient, adding them together, and then dividing them by two.

### 2.4 Statistical Analysis

The mean ± standard deviation is employed to represent continuous variables that follow a normal distribution, while the median and interquartile range (IQR) are used to present non‐normal distributions and are evaluated using nonparametric tests. The chi‐square test is employed to compare categorical variables, which are presented as frequencies (percentages). The minimal *p* value method was employed in the X‐tile software (version 3.6.1) to determine the optimal nutritional marker cutoffs. To determine whether the markers significantly predicted the outcome, we created Kaplan–Meier plots and compared them using the log‐rank test. Using the R survival package, univariate and multivariate Cox regression analyses were conducted to ascertain the independent prognostic significance of these indicators. Nomograms were constructed to predict the 0.5‐, 1‐, and 2‐year survival rates using the R rms package by employing the variables with independent prognostic significance. We used Harrell's concordance index (c‐index) and time‐dependent receiver operating characteristic curves to test the prediction accuracy of the nomogram. The calibration plot evaluated how well the predicted and observed values matched. Decision curve analysis (DCA) was used to determine whether the nomogram model was helpful in clinical settings. Statistical analysis was performed using R software (version 4.0.2; Institute for Statistics and Mathematics, Vienna, Austria). Statistical significance was defined as a two‐sided *p* value of < 0.05.

## 3 Results

### 3.1 Clinical Characteristics of Patients With GBM

As shown in Table S1, the baseline characteristics of the 309 participants revealed a median age of 59 years (IQR 52–66) with a predominance of male patients (58.58%). The median KPS score was 80 (IQR 60–80), and the median tumour volume was 34 cm^3^ (IQR 16–56). Most patients underwent GTR (77.35%), and a significant proportion underwent treatment with the Stupp protocol (71.20%). Additionally, most individuals had low MGMT promoter methylation status (54.45%). The median TMT was 7.80 mm (IQR 5.75–9.69). Various haematological and biochemical parameters, such as RBC and lymphocyte count and Hb, TP, and ALB levels were documented.

### 3.2 Prognostic Significance of Frailty Markers in Patients With GBM

**FIGURE 3:**
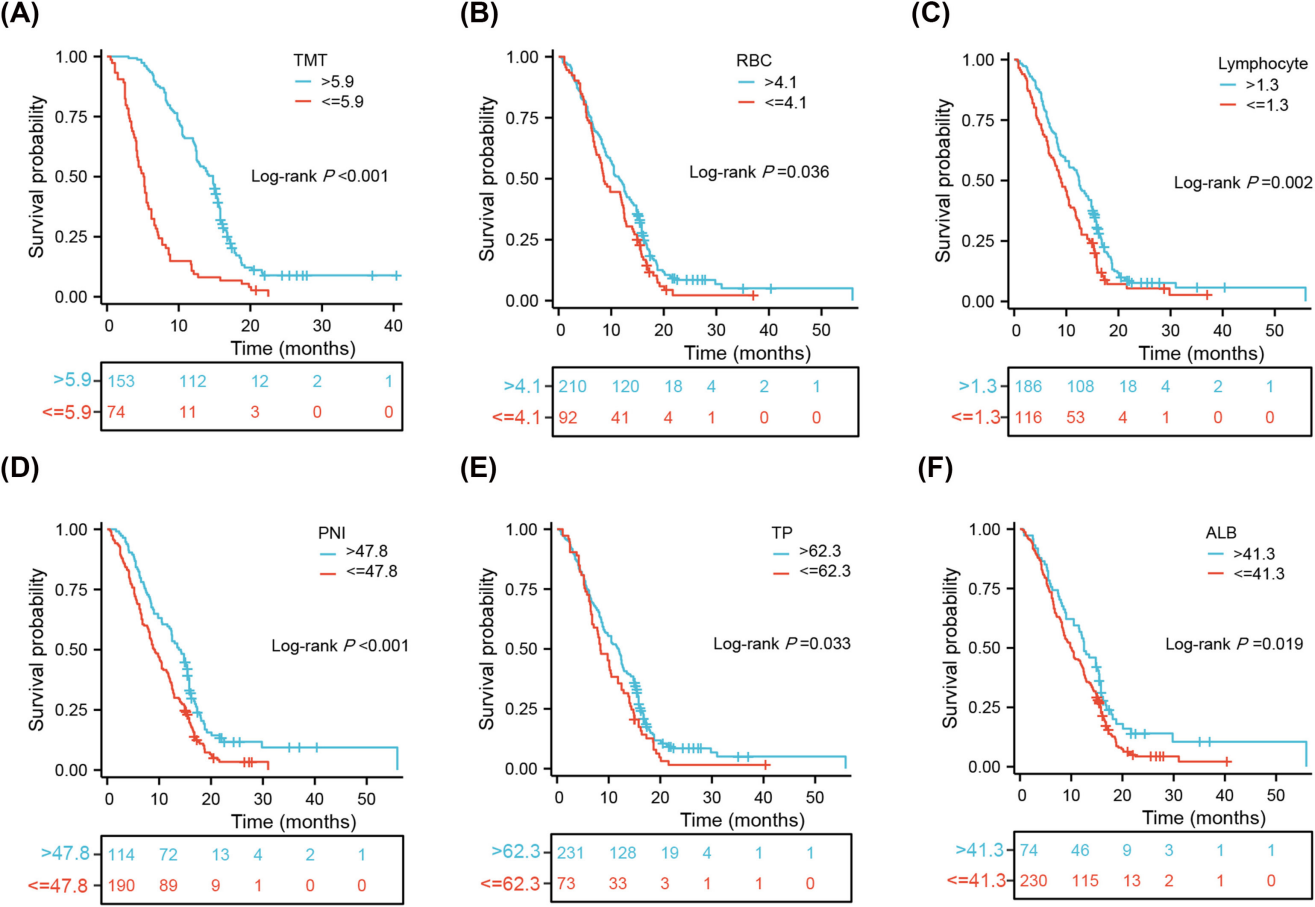
Kaplan–Meier survival curves of GBM patients based on the cutoff values of TMT (A), RBC (B), lymphocyte (C), PNI (D), TP (E), and ALB (F). GBM, glioblastoma; TMT, temporal muscle thickness; RBC, red blood cell; PNI, prognostic nutritional index; TP, total protein; ALB, albumin.

We determined the optimal threshold for each frail indicator using X‐tile tools. As seen in Figure S1, the cutoff values for TMT, BMI, RBC count, Hb level, lymphocyte count, HCT, PNI, MCV, MCH, MCHC, RDW, TP level, ALB level, GLB level, and AGR were 5.9 (mm), 19 (kg/m^2^), 4.1 (10^12^/L), 125.8 (g/L), 1.3 (10^9^/L), 38.3 (%), 47.8, 95.3 (fL), 31.5 (pg), 335.4 (g/L), 12.5 (%), 62.3 (g/L), 41.3 (g/L), 32.3 (g/L), and 1.8, respectively. Subsequently, patients were categorised into two groups according to the specific cutoff value for each marker. In the survival analysis, higher values of TMT (*p* < 0.001), RBC count (*p* = 0.036), lymphocyte count (*p* = 0.002), PNI (*p* < 0.001), TP level (*p* = 0.033), and ALB level (*p* = 0.019) were associated with improved outcomes (Figure 3). Conversely, older age (*p* = 0.009) and higher MCV (*p* = 0.009), and MCH (*p* = 0.008) were associated with worse OS (Figure S2). Indicators, including BMI, Hb level, HCT, MCHC, RDW, GLB level, and AGR, had no predictive significance (Figure S3). Multivariate analysis revealed TMT (*p* < 0.001), PNI (*p* = 0.018), and MCV (*p* = 0.020) as independent prognostic factors, alongside KPS, extent of tumour resection, postoperative standard chemoradiotherapy, and MGMT status (Table 1). These findings indicated that TMT, PNI, and MCV had independent prognostic values, whereas RBC count, lymphocyte count, TP level, ALB level, and MCH did not have such value. Furthermore, no significant associations were found among TMT, PNI, and MCV (Figure S4), suggesting that these three indicators reflect different aspects of an individual's frailty state.

**TABLE 1:** Univariate and multivariate analyses of OS in GBM cohorts.

Characteristics	Total (*N*)	Univariate analysis		Multivariate analysis	
		Hazard ratio (95% CI)	*p* value	Hazard ratio (95% CI)	*p* value
**Age**	309				
≤ 65	223	Reference		Reference	
> 65	86	1.418 (1.088–1.848)	**0.010**	1.262 (0.891–1.787)	0.190
**Sex**	309				
Male	181	Reference			
Female	128	0.945 (0.741–1.205)	0.648		
**KPS**	308	0.954 (0.947–0.962)	**< 0.001**	0.974 (0.963–0.985)	**< 0.001**
**Volume (cm** ^**3**^ **)**	235	1.000 (0.996–1.005)	0.895		
**Resection**	309				
GTR	239	Reference		Reference	
STR	70	4.208 (3.152–5.616)	**< 0.001**	2.149 (1.428–3.235)	**< 0.001**
**Stupp protocol completion**	309				
Yes	220	Reference		Reference	
No	89	11.179 (8.178–15.283)	**< 0.001**	6.848 (4.112–11.404)	**< 0.001**
**MGMT**	292				
Low	159	Reference		Reference	
High	133	0.751 (0.586–0.962)	**0.024**	0.582 (0.417–0.812)	**0.001**
**TMT**	227				
> 5.9	153	Reference		Reference	
≤ 5.9	74	3.546 (2.629–4.782)	**< 0.001**	3.787 (2.576–5.566)	**< 0.001**
**RBC**	302				
> 4.1	210	Reference		Reference	
≤ 4.1	92	1.314 (1.015–1.700)	**0.038**	0.702 (0.487–1.013)	0.059
**Lymphocyte**	302				
> 1.3	186	Reference		Reference	
≤ 1.3	116	1.491 (1.161–1.915)	**0.002**	0.895 (0.615–1.301)	0.561
**PNI**	304				
> 47.8	114	Reference		Reference	
≤ 47.8	190	1.639 (1.273–2.111)	**< 0.001**	1.722 (1.098–2.703)	**0.018**
**MCV**	302				
≤ 95.3	233	Reference		Reference	
> 95.3	69	1.447 (1.095–1.911)	**0.009**	1.958 (1.111–3.451)	**0.020**
**MCH**	302				
≤ 31.5	203	Reference		Reference	
> 31.5	99	1.407 (1.092–1.811)	**0.008**	0.715 (0.415–1.232)	0.227
**TP**	304				
> 62.3	231	Reference		Reference	
≤ 62.3	73	1.345 (1.023–1.768)	**0.033**	0.861 (0.596–1.243)	0.424
**ALB**	304				
> 41.3	74	Reference		Reference	
≤ 41.3	230	1.408 (1.055–1.879)	**0.020**	0.978 (0.618–1.550)	0.925

Abbreviations: ALB, albumin; CI, confidence interval; GTR, gross total resection; KPS, Karnofsky performance scale; MCH, mean corpuscular haemoglobin; MCV, mean corpuscular volume; MGMT, O6‐methylguanine‐DNA methyltransferase; PNI, prognostic nutritional index; RBC, red blood cell; STR, subtotal resection; TMT, temporal muscle thickness; TP, total protein.

### 3.3 Prognostic Significance of Modified Frailty Score in Patients With GBM

**FIGURE 4:**
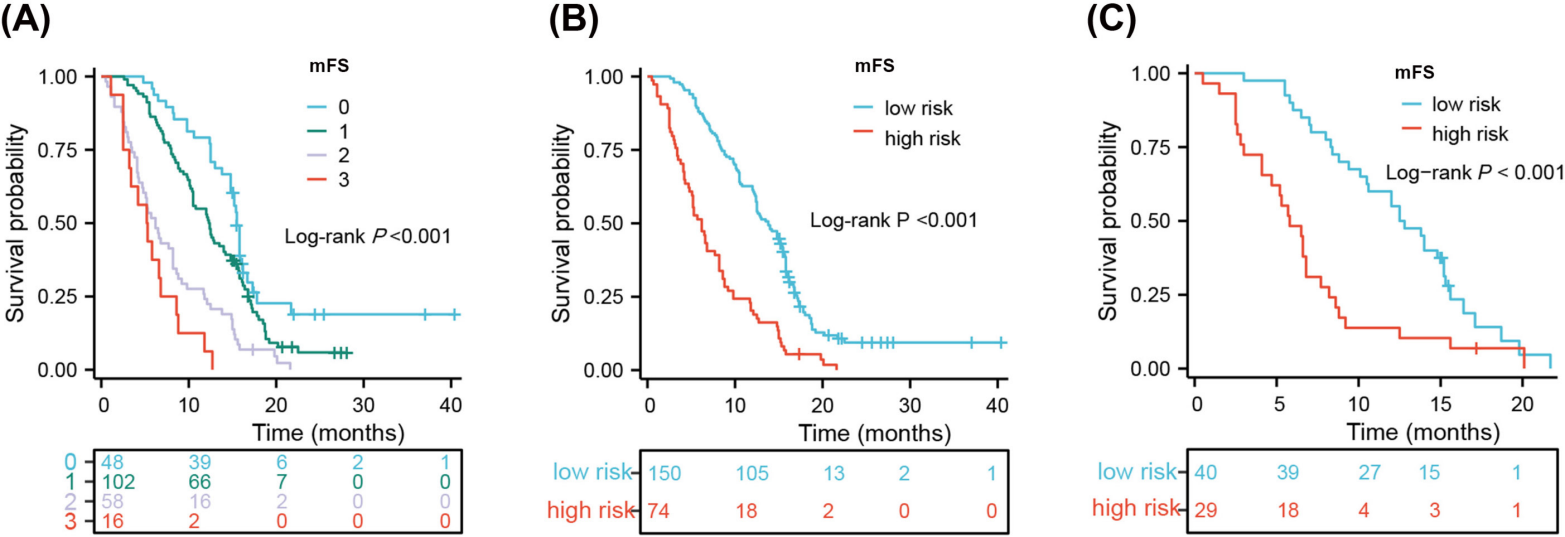
Kaplan–Meier survival curves based on the mFS in the overall (A,B) and elderly populations (C) of GBM. mFS, modified frailty score; GBM, glioblastoma.

In light of these findings, TMT, PNI, and MCV were combined to form the modified frailty score (mFS). The negative association between each marker's status and survival outcome (TMT ≤ 5.9, PNI ≤ 47.8, MCV > 95.3) was used to determine the mFS (Table S2). A score of 0 indicated no variables present in the mFS, 1 indicated the presence of one variable, 2 indicated two variables, and 3 indicated all three variables. Figure 4A shows a significant association between the mFS and OS in patients with GBM. However, further subgroup analysis indicated that there was no significant difference between the score 0 group and the score 1 group, nor between the score 2 group and the score 3 group (Table S3). Therefore, we further divided the mFS into two groups: low‐risk (scores 0 and 1) and high‐risk (scores 2 and 3) (Table S2). The new mFS risk group exhibited independent prognostic value in patients with GBM (HR = 2.461, 95% CI 1.751–3.457, *p* < 0.001; Figure 4B, Table 2); the demographic characteristics based on the mFS risk group are shown in Table S4. The patients in the low‐risk group had a median OS of 13.9 months, while the patients in the high‐risk had a median OS of 5.8 months (Table S4). Based on the extent of tumour resection, we also performed corresponding subgroup analyses, and the results indicated that mFS manifested significant prognostic value in both the GTR group and the STR group (Figure S5). Considering that older patients with GBM (> 65 years) may experience poorer frailty status and that attention paid to the frailty status was relatively lacking, we further analysed the prognostic value of the mFS in the subgroup of patients aged ≥65 years, and the mFS exhibited independent prognostic significance (HR = 1.822, 95% CI 1.011–3.284, *p* = 0.046) (Figure 4C, Table 3).

**TABLE 2:** Univariate and multivariate analyses of OS based on mFS in GBM cohorts.

Characteristics	Total (*N*)	Univariate analysis		Multivariate analysis	
		Hazard ratio (95% CI)	*p* value	Hazard ratio (95% CI)	*p* value
**Age**	309				
≤ 65	223	Reference		Reference	
> 65	86	1.418 (1.088–1.848)	**0.010**	1.114 (0.797–1.557)	0.528
**Sex**	309				
Male	181	Reference			
Female	128	0.945 (0.741–1.205)	0.648		
**KPS**	308	0.954 (0.947–0.962)	**< 0.001**	0.979 (0.968–0.990)	**< 0.001**
**Volume (cm** ^**3**^ **)**	235	1.000 (0.996–1.005)	0.895		
**Resection**	309				
GTR	239	Reference		Reference	
STR	70	4.208 (3.152–5.616)	**< 0.001**	1.994 (1.334–2.981)	**< 0.001**
**Stupp protocol completion**	309				
Yes	220	Reference		Reference	
No	89	11.179 (8.178–15.283)	**< 0.001**	6.362 (4.069–9.948)	**< 0.001**
**MGMT**	292				
Low	159	Reference		Reference	
High	133	0.751 (0.586–0.962)	**0.024**	0.722 (0.534–0.976)	**0.034**
**mFS**	224				
Low risk	150	Reference		Reference	
High risk	74	2.895 (2.151–3.897)	**< 0.001**	2.461 (1.751–3.457)	**< 0.001**

Abbreviations: CI, confidence interval; GTR, gross total resection; KPS, Karnofsky performance scale; MGMT, O6‐methylguanine‐DNA methyltransferase; mFS, modified frailty score; STR, subtotal resection.

**TABLE 3:** Univariate and multivariate analyses of OS in older patients with GBM.

Characteristics	Total (*N*)	Univariate analysis		Multivariate analysis	
		Hazard ratio (95% CI)	*p* value	Hazard ratio (95% CI)	*p* value
**Sex**	86				
Male	53	Reference			
Female	33	1.437 (0.903–2.288)	0.127		
**KPS**	86	0.963 (0.950–0.977)	**< 0.001**	0.983 (0.963–1.003)	0.094
**Volume (cm3)**	69	1.003 (0.994–1.011)	0.553		
**Resection**	86				
GTR	66	Reference		Reference	
STR	20	6.382 (3.448–11.812)	**< 0.001**	2.269 (1.036–4.966)	**0.040**
**Stupp protocol completion**	86				
Yes	56	Reference		Reference	
No	30	9.127 (5.182–16.076)	**< 0.001**	5.984 (2.939–12.184)	**< 0.001**
**MGMT**	81				
Low	40	Reference			
High	41	0.701 (0.441–1.113)	0.132		
**mFS**	69				
Low risk	40	Reference		Reference	
High risk	29	2.624 (1.562–4.408)	**< 0.001**	1.822 (1.011–3.284)	**0.046**

Abbreviations: GTR, gross total resection; KPS, Karnofsky performance scale; MGMT, O6‐methylguanine‐DNA methyltransferase; mFS, modified frailty score; STR, subtotal resection.

### 3.4 Prognostic Nomograms for Assessing OS in Patients With GBM

**FIGURE 5:**
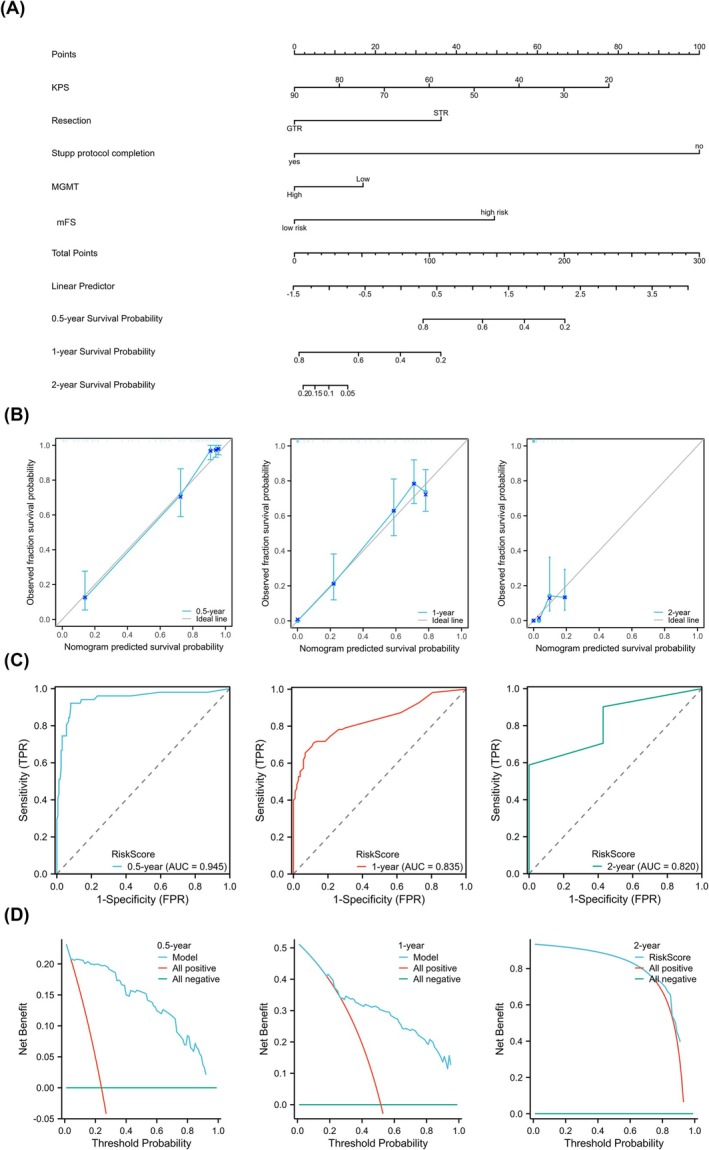
Nomogram, calibration curve, time‐dependent ROC curves and DCA. A numerical forecast of 0.5‐, 1‐, or 2‐year survival was obtained by adding up the scores of all the variables in the nomogram (A), where greater scores indicated a poorer prognosis. The scores were shown on the biggest scale, and each variable was given a unique value. The blue line in the calibration curve (B) shows the nomogram's performance, whereas the grey line represents the ideal forecast. (C) The area under ROC curve was used to judge the accuracy of the nomogram. (D) DCA curve for the prognostic nomogram. The benefit net, which is the result of combining all true positives and minus all false positives, is shown on the *Y*‐axis, while the threshold probability is shown on the *X*‐axis. ROC, receiver operating characteristic; DCA, decision curve analysis.

Oncology researchers often employ nomograms, which are visual calculation scale models, to estimate digital prognosis by effectively integration of several prognostic factors. Consequently, a nomogram combining KPS, extent of resection, chemoradiotherapy, MGMT, and the mFS was developed to examine the potential of these factors in predicting 0.5‐, 1‐, and 2‐year survival in patients with GBM (Figure 5A). Chemoradiotherapy accounted for the most significant weightage in the nomogram, followed by KPS, mFS, resection, and MGMT. The c‐index of the nomogram was 0.781 (95% confidence interval [CI] = 0.762–0.799). A better performance was achieved in the bootstrapped calibration plot of the nomogram compared with the ideal model (Figure 5B). Nomograms showed promising discriminative potential, with time‐dependent areas under the curves of 0.945, 0.835, and 0.820 for the 0.5‐, 1‐, and 2‐year OS predictions, respectively (Figure 5C). Additionally, DCA has been extensively used to determine the clinical significance of nomograms. The nomogram revealed a considerable positive net benefit from the risk of mortality, as shown in Figure 5D. These findings demonstrate the outstanding practical and clinical usefulness of the nomogram for predicting OS in patients with GBM.

## 4 Discussion

Frailty is a well‐known and common issue in surgical oncology, associated with poor postoperative outcomes, quality of life, and overall prognosis. Despite being a modifiable clinical feature, frailty status is often disregarded, partly because there is no universally accepted method for its evaluation. Numerous new and practical frailty indicators, including PNIs, have been reported. Our retrospective analysis confirmed that PNI, MCV, and TMT are independent predictive indicators in patients with GBM. An innovative marker, the mFS, was developed by combining PNI, MCV, and TMT. It reflects an individual's frailty status from haematological and sarcopenia aspects and has independent prognostic significance. Additionally, the established nomogram that included the mFS had great clinical practical value.

The link between tumours and malnutrition has become a popular topic. Multiple malignancies have been associated with a poor prognosis in patients with nutritional deficiencies. Serum albumin concentration and lymphocyte count are used to determine PNI, which represents the immune‐nutritional state. PNI is a predictive marker in various cancer types, including oesophageal cancer [24], and gastrointestinal cancer [10]. Moreover, it has been reported as an independent predictor of OS and the effectiveness of adjuvant treatment in patients with GBM [25]. While a study has reported that a greater PNI is associated with a longer OS in patients with GBM, it does not confirm that PNI is an independent prognostic factor [26]. MCV, which measures the variation in RBC volume and differentiates between different types of anaemia, has been utilised as a host nutritional index for the predicting long‐term outcomes in patients with various cancers, such as oesophageal cancer [27], and gastroesophageal adenocarcinoma [28]. However, the prognostic and predictive values of MCV in GBM may have been overlooked in the past. Another frailty marker, sarcopenia, has been linked to a poor prognosis in cancers [29]. Increasingly, researchers are investigating TMT to determine whether it could substitute for sarcopenia as a survival predictor in patients with GBM. Recently, researchers also noted that TMT could serve as a reference indicator of prognosis in patients with newly diagnosed GBM, with a thinner TMT indicating a worse prognosis [30]. Nevertheless, whether TMT can be used independently as a predictor of survival in patients with GBM remains debatable. In newly diagnosed GBM cases, Wende et al. found that TMT could not be used as an independent predictive factor [31]. In this study, we found that patients with GBM who had lower MCVs, higher PNI, or TMT had better outcomes. Since no discernible relationship exists between PNI, TMT, and MCV, the mFS that incorporates all three indicators may offer a comprehensive assessment of an individual's frailty status. The mFS risk group had independent prognostic significance (HR = 2.461, 95% CI 1.751–3.457), underscoring its significant clinical practical value in the nomogram model.

The exact mechanisms by which these frailty indicators affect the prognosis of patients with GBM are yet unknown. PNI, calculated using the albumin level and lymphocyte count, has the potential to significantly affect the prognosis of patients with cancer. Cytokines and growth factors released by cancer cells or systemic inflammatory reactions caused by tumours may influence albumin production in the liver. The role of lymphocytes in cell‐mediated immune responses to cancer is well established, and it is believed that the inhibition of tumour migration and proliferation may be achieved by activating and recruiting lymphocytes within the tumour microenvironment [32]. Conversely, glioma cells may restrict lymphocyte activation and recruitment by suppressing dendritic cell development and activation, tumour‐associated macrophages or neutrophils, and the consequent infiltration of regulatory T cells [33]. Therefore, low PNI may reflect lymphocytopenia and hypoalbuminemia, both signs of poor prognosis in patients with GBM. As tumour stroke, inadequate nutrition, and chronic inflammation are causes of anaemia, MCV may function as a marker for this condition, which is associated with host nutrition. This is important since anaemia is a symptom of cancer. An earlier study proposed that hypoxia in the tumour microenvironment, induced by chronic blood loss and malnutrition, increases hypoxia‐inducible factor‐1a expression and T‐cell apoptosis, consequently reducing total lymphocyte levels and facilitating tumour revascularisation and proliferation [34]. Significantly, the dynamic changes of MCV may serve as predictive markers for chemotherapy efficacy and survival outcomes in patients with advanced gastric cancer [35]. Previous studies have shown that sarcopenia is associated with poor prognosis in patients with cancer [12], and TMT may be used as an alternative measure of skeletal muscle mass for diagnosing sarcopenia [13]. A decrease in skeletal muscle mass attributed to aging is often referred to as primary sarcopenia, whereas secondary sarcopenia is associated with many detrimental events, such as illnesses. Additional components of the frailty phenotype, including dietary state, skeletal muscle mass, functional state, and medical comorbidities, are associated with secondary sarcopenia [36]. Frailty, a physiological condition of diminished reserve linked to an increased likelihood of adverse clinical outcomes, is a well‐known reason for poor postoperative results in patients undergoing surgery, including those with brain tumours [37]. Therefore, sarcopenia is often used as a component of frailty in clinical settings to evaluate patient prognosis. It increases the likelihood of worse survival in patients with cancers [29, 38]. Several fragility indicators, including grip strength, food preferences, and functional capacity, have recently been linked to TMT. Hence, similar to how sarcopenia is used in other branches of surgery, TMT may be included in a thorough initial frailty evaluation in neurosurgical oncology to evaluate patient prognosis. Moreover, patients undergoing severe neurological surgery demonstrate diminished muscle activity compared to other critically ill individuals, potentially leading to significant loss of lean body mass within a relatively short period. This muscle atrophy is linked to prolonged hospitalisation and can negatively impact functional outcomes while increasing mortality rates. Therefore, in these patients, the assessment of frailty status should prioritise the evaluation of muscle quality. The TMT, recognised as an innovative metric for assessing muscle quality, has been correlated closely with overall muscular health and has independent prognostic significance in various cancer populations, including those with GBM. Therefore, this study incorporated TMT into the final mFS score to accurately reflect the muscle mass of GBM patients and facilitate a more comprehensive frailty assessment. Notably, patients with sarcopenic GBM have a much lower likelihood of receiving second‐line treatment in case of recurrence and a substantially higher likelihood of prematurely discontinuing Stupp therapy [39]. One potential explanation is that sarcopenic patients frequently exhibit compromised overall physical function, which may hinder their adherence to standard treatment protocols and result in reduced tolerance for adverse reactions associated with these therapies.

This study has innovatively introduced a comprehensive frailty assessment tool known as mFS, grounded in haematological and sarcopenia indicators. The findings indicate that elevated mFS scores correlate with poorer prognoses in GBM patients, thereby underscoring the critical role of frailty assessment in the clinical management of neurological tumours. By stratifying patients according to their baseline frailty assessment levels, this research offers valuable insights for clinicians and healthcare providers. For those identified with low frailty risk, more aggressive treatment modalities such as surgery and chemoradiotherapy may be pursued. Conversely, for patients at high frailty risk, treatment strategies might necessitate a more conservative approach. Furthermore, an online tool designed for assessing GBM survival rates was developed, significantly enhancing the clinical applicability of the nomogram (https://yangchao.shinyapps.io/gbmnutrition/). Looking ahead, if electronic medical record systems can automatically compute mFS and integrate these results into the nomogram model, clinicians will be better equipped to promptly assess their patients' frailty and make informed decisions regarding suitable treatment options.

Our study has some limitations. First, the limited sample size and retrospective design may introduce selection bias. Second, certain data items (such as MRI and molecular pathological markers) were absent for several people. Third, interpretation of some selected serum markers also warrants caution. Serum albumin and total lymphocyte count have traditionally served as frailty markers; however, they are now primarily recognised for their role in reflecting systemic inflammation and immune status associated with nutritional risk [40]. Moreover, it is inaccurate to classify sarcopenia as a frailty indicator since this condition results from various factors such as nutrient deficiencies, reduced physical activity, and oxidative stress. Furthermore, this study exclusively examined the significance of preoperative baseline frailty indicators in prognosis assessment, without investigating the influence of tumour progression or variations in these indicators during treatment on prognostic and predictive evaluations. Additionally, frailty status is closely associated with nutritional status. The standardisation of nutritional monitoring and diagnostic tools is crucial for both scientific research and clinical practice. Established tools such as the mini nutritional assessment‐short form (MNA‐SF), malnutrition universal screening tool (MUST), global leadership initiative on malnutrition (GLIM) criteria, and full MNA have shown high reliability and reproducibility in assessing nutrition across various diseases. However, in our study, some patients lacked data of these standardised nutritional assessment. In the future, it is necessary to incorporate standardised nutritional risk assessment and diagnostic tools to achieve a comprehensive nutritional evaluation for GBM patients. Thus, prospective comparative studies with strict inclusion and exclusion criteria are required to confirm our findings and guide clinical management. Additional research on frailty assessment, an underexplored area in neurosurgical oncology, is needed. Preoperative optimisation clinics, focusing on improving the patients' total functional ability and reserves before tumour surgery, could become widely used in the future.

## 5 Conclusions

Preoperative mFS is a comprehensive frailty marker for predicting survival outcomes in patients with GBM. A dynamic nomogram incorporating the mFS may facilitate preoperative survival evaluation. Early and appropriate multimodal interventions, including nutritional support, rehabilitation, and psychological care, may help in the neurosurgical care of patients with GBM or other brain tumours.

## Ethics Statement

The authors of this manuscript certify that they comply with the ethical guidelines for authorship and publishing in the Journal of Cachexia, Sarcopenia and Muscle. The study adhered to the principles outlined in the Declaration of Helsinki and the Ethics Committee of Zhongnan Hospital of Wuhan University approved the present study (no. 2019048). The informed consent from all the included patients was unnecessary due to its retrospective nature. The relevant information was coded and the patients were de‐identified.

## Conflicts of Interest

The authors declare no conflicts of interest.

## Supporting information


**Figure S1** Cutoff value of TMT, BMI, and RBC (A); Hb, Lymphocyte and HCT (B); PNI, MCV, and MCH (C); MCHC, RDW, and TP (D); ALB, GLB, and AGR (E) in patients with GBM. TMT, temporal muscle thickness; BMI, body mass index; RBC, red blood cell; Hb, haemoglobin; HCT, haematocrit; PNI, prognostic nutritional index; MCV, mean corpuscular volume; MCH, mean corpuscular haemoglobin; MCHC, mean corpuscular haemoglobin concentration; RDW, RBC distribution width; TP, total protein; ALB, albumin; GLB, globulin; AGR, albumin‐to‐globulin ratio; GBM, glioblastoma.

**Figure S2** Kaplan–Meier survival curves of GBM patients based on the cutoff values of Age (A), MCV (B) and MCH (C). GBM, glioblastoma; MCV, mean corpuscular volume; MCH, mean corpuscular haemoglobin.

**Figure S3** Kaplan–Meier survival curves of GBM patients based on the cutoff values of BMI, Hb, and HCT (A); MCHC, RDW, and GLB (B); AGR (C). GBM, glioblastoma; BMI, body mass index; Hb, haemoglobin; HCT, haematocrit; MCHC, mean corpuscular haemoglobin concentration; RDW, RBC distribution width; GLB, globulin; AGR, albumin‐to‐globulin ratio.

**Figure S4** Correlation analysis of TMT, PNI, and MCV. TMT, temporal muscle thickness; PNI, prognostic nutritional index; MCV, mean corpuscular volume.

**Figure S5** Kaplan–Meier survival curves based on the mFS risk groups in GBM patients with gross total resection (A) and subtotal resection (B). mFS, modified frailty score; GBM, glioblastoma.

**Table S1**Patient demographics and baseline characteristics.

**Table S2** Definition of mFS risk stratification in patients with GBM.Abbreviations: mFS, modified frailty score; TMT, temporal muscle thickness; PNI, prognostic nutritional index; MCV, mean corpuscular volume; OS, overall survival; SD, standard deviation.

**Table S3** Pairwise comparisons based on different scores of mFS.Abbreviations: HR, hazard ratio; CI, confidence interval.

**Table S4** Patient demographics and baseline characteristics based on mFS risk group. Abbreviations: KPS, Karnofsky performance scale; GTR, gross total resection; STR, subtotal resection; MGMT, O6‐methylguanine‐DNA methyltransferase; TMT, temporal muscle thickness; ALB, albumin; PNI, prognostic nutritional index; MCV, mean corpuscular volume; OS, overall survival; mFS, modified frailty score.

**Data S1** Supplementary information.

## Data Availability

The original dataset analysed during the current study is available from the corresponding author upon reasonable request.
