# Supplementary figures and images for: Comprehensive Evaluation of Frailty and Sarcopenia Markers to Predict Survival in Glioblastoma Patients
Source: J Cachexia Sarcopenia Muscle. 2025 Apr 15;16(2):e13809. doi: 10.1002/jcsm.13809 (PMC11999731; doi:10.1002/jcsm.13809)

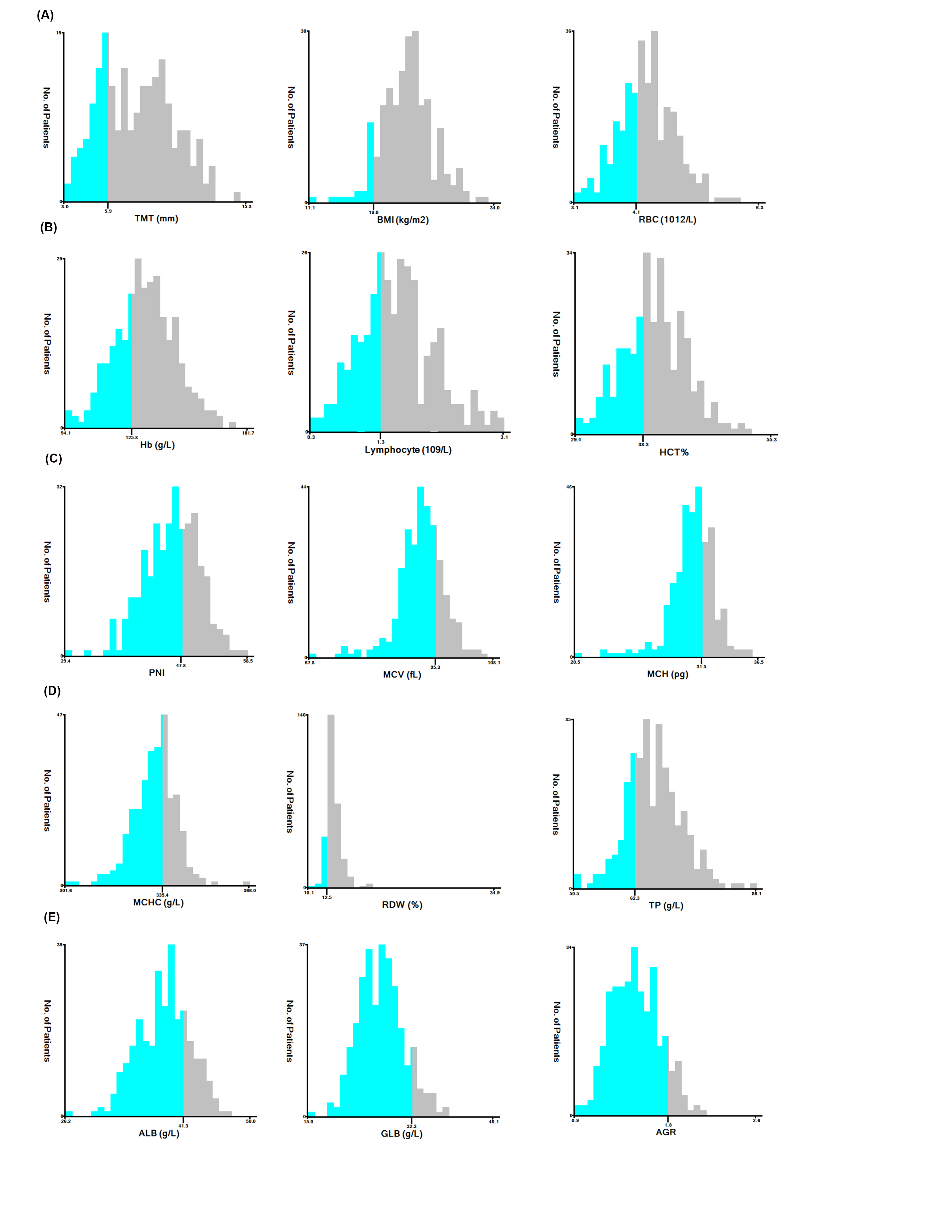

Supplement: Supplementary file 1 — Figure S1 Cutoff value of TMT, BMI, and RBC (A); Hb, Lymphocyte and HCT (B); PNI, MCV, and MCH (C); MCHC, RDW, and TP (D); ALB, GLB, and AGR (E) in patients with GBM. TMT, temporal muscle thickness; BMI, body mass index; RBC, red blood cell; Hb, haemoglobin; HCT, haematocrit; PNI, prognostic nutritional index; MCV, mean corpuscular volume; MCH, mean corpuscular haemoglobin; MCHC, mean corpuscular haemoglobin concentration; RDW, RBC distribution width; TP, total protein; ALB, albumin; GLB, globulin; AGR, albumin‐to‐globulin ratio; GBM, glioblastoma. [file JCSM-16-e13809-s001.tif]

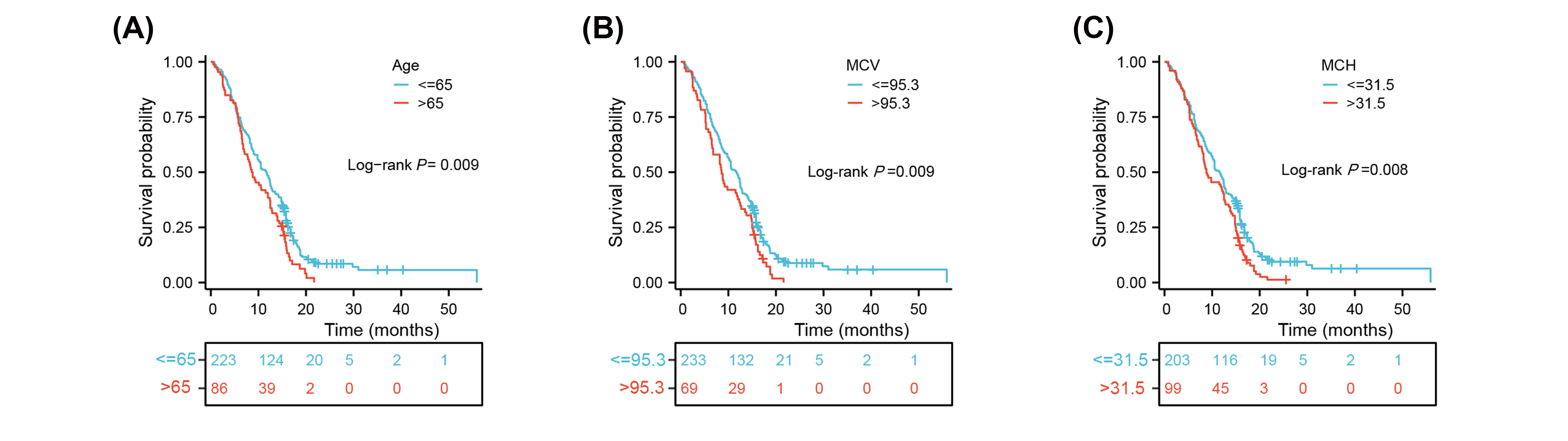

Supplement: Supplementary file 2 — Figure S2 Kaplan–Meier survival curves of GBM patients based on the cutoff values of Age (A), MCV (B) and MCH (C). GBM, glioblastoma; MCV, mean corpuscular volume; MCH, mean corpuscular haemoglobin. [file JCSM-16-e13809-s010.tif]

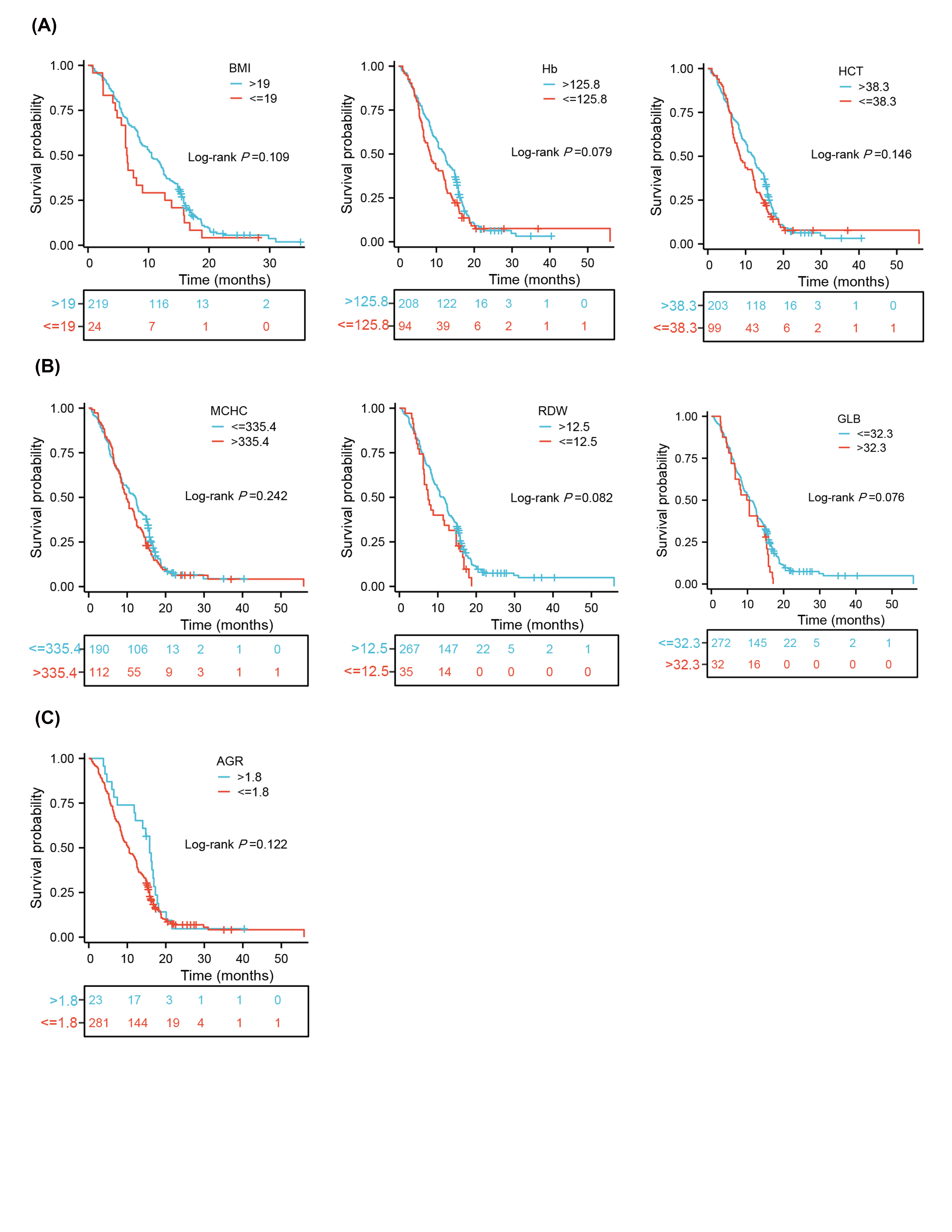

Supplement: Supplementary file 3 — Figure S3 Kaplan–Meier survival curves of GBM patients based on the cutoff values of BMI, Hb, and HCT (A); MCHC, RDW, and GLB (B); AGR (C). GBM, glioblastoma; BMI, body mass index; Hb, haemoglobin; HCT, haematocrit; MCHC, mean corpuscular haemoglobin concentration; RDW, RBC distribution width; GLB, globulin; AGR, albumin‐to‐globulin ratio. [file JCSM-16-e13809-s008.tif]

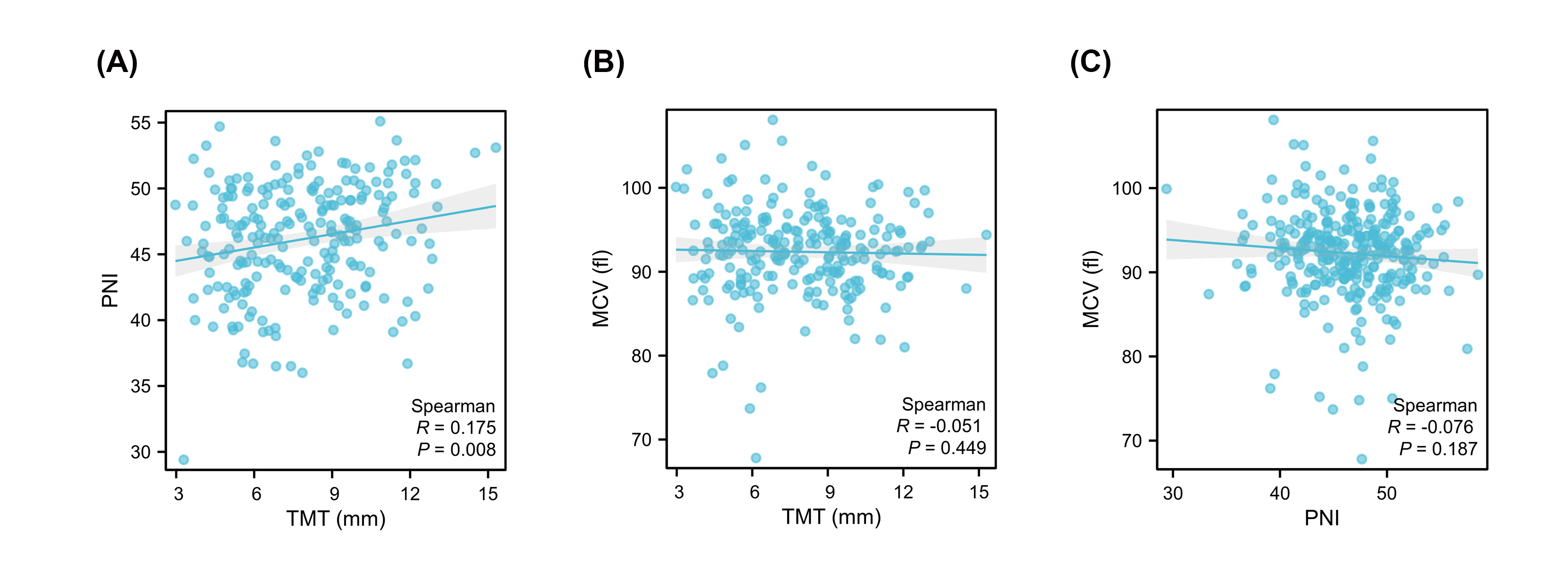

Supplement: Supplementary file 4 — Figure S4 Correlation analysis of TMT, PNI, and MCV. TMT, temporal muscle thickness; PNI, prognostic nutritional index; MCV, mean corpuscular volume. [file JCSM-16-e13809-s006.tif]

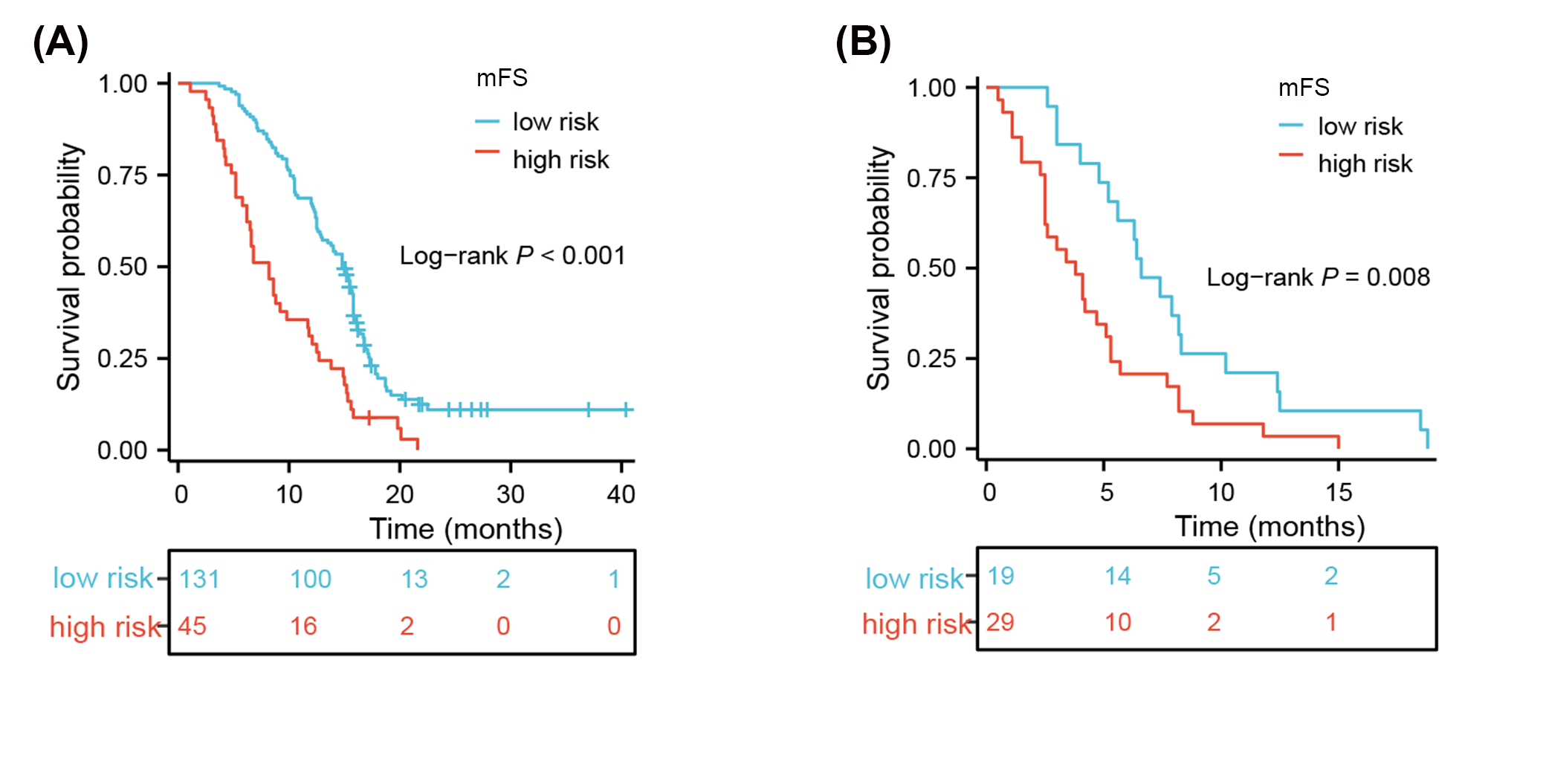

Supplement: Supplementary file 5 — Figure S5 Kaplan–Meier survival curves based on the mFS risk groups in GBM patients with gross total resection (A) and subtotal resection (B). mFS, modified frailty score; GBM, glioblastoma. [file JCSM-16-e13809-s005.tif]
