# Supplementary material for: Comprehensive Evaluation of Frailty and Sarcopenia Markers to Predict Survival in Glioblastoma Patients
Source: J Cachexia Sarcopenia Muscle. 2025 Apr 15;16(2):e13809. doi: 10.1002/jcsm.13809 (PMC11999731; doi:10.1002/jcsm.13809)
Supplement: Supplementary file 6 — Table S1Patient demographics and baseline characteristics. [file JCSM-16-e13809-s009.docx]

**Supplementary Table S1.** Patient demographics and baseline characteristics

| **Characteristics** | **N = 309^1^** |
| --- | --- |
| **Age** | 59 (52, 66) |
| **Sex** |  |
| female | 128 (41.42%) |
| male | 181 (58.58%) |
| **KPS** | 80 (60, 80) |
| **Volume（cm3）** | 34 (16, 56) |
| **Resection** |  |
| STR | 70 (22.65%) |
| GTR | 239 (77.35%) |
| **Stupp protocol completion** |  |
| no | 89 (28.80%) |
| yes | 220 (71.20%) |
| **MGMT** |  |
| Low | 159 (54.45%) |
| High | 133 (45.55%) |
| Unknown | 17 |
| **TMT** | 7.80 (5.75, 9.69) |
| **BMI** | 23.2 ± 3.3 |
| **RBC** | 4.30 (4.01, 4.65) |
| **Hb** | 133 ± 15 |
| **Lymphocyte** | 1.50 (1.16, 1.80) |
| **HCT** | 39.9 ± 4.3 |
| **PNI** | 46.4 ± 4.4 |
| **MCV** | 92.6 (89.5, 95.0) |
| **MCH** | 30.90 (29.80, 31.80) |
| **MCHC** | 334 (329, 337) |
| **RDW** | 13.30 (12.90, 13.80) |
| **TP** | 65.6 (62.4, 69.3) |
| **ALB** | 38.8 ± 3.4 |
| **GLB** | 27.1 (24.4, 29.7) |
| **AGR** | 1.43 (1.28, 1.61) |
| **OS** | 10.5 (5.8, 15.5) |
| ^1^Median (IQR); n (%); Mean ± SD. Abbreviations: KPS, Karnofsky performance scale; GTR, gross total resection; STR, subtotal resection; MGMT, O6-methylguanine-DNA methyltransferase; TMT, temporal muscle thickness; BMI, body mass index; RBC, red blood cell; Hb, hemoglobin; HCT, hematocrit; PNI, prognostic nutritional index; MCV, mean corpuscular volume; MCH, mean corpuscular hemoglobin; MCHC, mean corpuscular hemoglobin concentration; RDW, red blood cell distribution width; TP, total protein; ALB, albumin; GLB, globulin; AGR, albumin-to-globulin ratio; OS, overall survival. | |
