# Supplementary material for: Comprehensive Evaluation of Frailty and Sarcopenia Markers to Predict Survival in Glioblastoma Patients
Source: J Cachexia Sarcopenia Muscle. 2025 Apr 15;16(2):e13809. doi: 10.1002/jcsm.13809 (PMC11999731; doi:10.1002/jcsm.13809)
Supplement: Supplementary file 7 — Table S2 Definition of mFS risk stratification in patients with GBM.Abbreviations: mFS, modified frailty score; TMT, temporal muscle thickness; PNI, prognostic nutritional index; MCV, mean corpuscular volume; OS, overall survival; SD, standard deviation. [file JCSM-16-e13809-s004.docx]

**Supplementary Table S2.** Definition of mFS risk stratification in patients with GBM

| **mFS** | **Definition** | **Risk stratification** |
| --- | --- | --- |
| **Score 0** | TMT>5.9 and PNI>47.8 and MCV≤95.3 | **Low risk** |
| **Score 1** | TMT≤5.9 or PNI≤47.8 or MCV>95.3 | **Low risk** |
| **Score 2** | TMT≤5.9 and PNI≤47.8, or TMT≤5.9 and MCV>95.3, or PNI≤47.8 and MCV>95.3 | **High risk** |
| **Score 3** | TMT≤5.9 and PNI≤47.8 and MCV>95.3 | **High risk** |

Abbreviations: mFS, modified frailty score; TMT, temporal muscle thickness; PNI, prognostic nutritional index; MCV, mean corpuscular volume; OS, overall survival; SD, standard deviation.
