# Supplementary material for: Comprehensive Evaluation of Frailty and Sarcopenia Markers to Predict Survival in Glioblastoma Patients
Source: J Cachexia Sarcopenia Muscle. 2025 Apr 15;16(2):e13809. doi: 10.1002/jcsm.13809 (PMC11999731; doi:10.1002/jcsm.13809)
Supplement: Supplementary file 8 — Table S3 Pairwise comparisons based on different scores of mFS.Abbreviations: HR, hazard ratio; CI, confidence interval. [file JCSM-16-e13809-s002.docx]

**Supplementary Table S3.** Pairwise comparisons based on different scores of mFS

| **Group 1** | **Group 2** | **HR** | **CI** | **p** | **Adjusted p** |
| --- | --- | --- | --- | --- | --- |
| Score 0 | Score 1 | 1.581 | 1.097-2.279 | 0.020 | 0.121 |
| Score 0 | Score 2 | 3.209 | 2.082-4.947 | <0.001 | <0.001 |
| Score 0 | Score 3 | 6.371 | 2.131-19.050 | <0.001 | <0.001 |
| Score 1 | Score 2 | 2.137 | 1.454-3.141 | <0.001 | <0.001 |
| Score 1 | Score 3 | 4.448 | 1.654-11.964 | <0.001 | <0.001 |
| Score 2 | Score 3 | 1.559 | 0.824-2.951 | 0.101 | 0.604 |

Abbreviations: HR, hazard ratio; CI, confidence interval.
