# Supplementary material for: Comprehensive Evaluation of Frailty and Sarcopenia Markers to Predict Survival in Glioblastoma Patients
Source: J Cachexia Sarcopenia Muscle. 2025 Apr 15;16(2):e13809. doi: 10.1002/jcsm.13809 (PMC11999731; doi:10.1002/jcsm.13809)
Supplement: Supplementary file 9 — Table S4 Patient demographics and baseline characteristics based on mFS risk group. Abbreviations: KPS, Karnofsky performance scale; GTR, gross total resection; STR, subtotal resection; MGMT, O6‐methylguanine‐DNA methyltransferase; TMT, temporal muscle thickness; ALB, albumin; PNI, prognostic nutritional index; MCV, mean corpuscular volume; OS, overall survival; mFS, modified frailty score. [file JCSM-16-e13809-s007.docx]

**Supplementary Table S4**. Patient demographics and baseline characteristics based on mFS risk group

| **Characteristics** | **mFS** | | **p-value** |
| --- | --- | --- | --- |
|  | **low risk, N = 150^1^** | **high risk, N = 74** |  |
| **Age** |  |  | 0.027 |
| <=65 | 111 (74.00%) | 44 (59.46%) |  |
| >65 | 39 (26.00%) | 30 (40.54%) |  |
| **Sex** |  |  | 0.910 |
| female | 62 (41.33%) | 30 (40.54%) |  |
| male | 88 (58.67%) | 44 (59.46%) |  |
| **KPS** | 80 (70, 90) | 70 (50, 80) | <0.001 |
| Unknown | 0 | 1 |  |
| **Volume（cm3）** | 33 (15, 55) | 36 (18, 58) | 0.397 |
| Unknown | 5 | 0 |  |
| **Resection** |  |  | <0.001 |
| GTR | 131 (87.33%) | 45 (60.81%) |  |
| STR | 19 (12.67%) | 29 (39.19%) |  |
| **Chemoradiotherapy** |  |  | <0.001 |
| yes | 126 (84.00%) | 37 (50.00%) |  |
| no | 24 (16.00%) | 37 (50.00%) |  |
| **MGMT** |  |  | 0.291 |
| High | 63 (43.75%) | 36 (51.43%) |  |
| Low | 81 (56.25%) | 34 (48.57%) |  |
| Unknown | 6 | 4 |  |
| **TMT** |  |  | <0.001 |
| >5.9 | 134 (89.33%) | 18 (24.32%) |  |
| <=5.9 | 16 (10.67%) | 56 (75.68%) |  |
| **Lymphocyte** | 1.60 (1.30, 1.91) | 1.30 (0.93, 1.72) | <0.001 |
| **ALB** | 39.2 ± 3.3 | 37.1 ± 3.1 | <0.001 |
| **PNI** |  |  | <0.001 |
| <=47.8 | 73 (48.67%) | 68 (91.89%) |  |
| >47.8 | 77 (51.33%) | 6 (8.11%) |  |
| **MCV** |  |  | <0.001 |
| <=95.3 | 137 (91.33%) | 34 (45.95%) |  |
| >95.3 | 13 (8.67%) | 40 (54.05%) |  |
| **OS** | 13.9 (8.8, 16.1) | 5.8 (3.4, 9.7) | <0.001 |

Abbreviations: KPS, Karnofsky performance scale; GTR, gross total resection; STR, subtotal resection; MGMT, O6-methylguanine-DNA methyltransferase; TMT, temporal muscle thickness; ALB, albumin; PNI, prognostic nutritional index; MCV, mean corpuscular volume; OS, overall survival; mFS, modified frailty score.
