# Supplementary material for: Comprehensive Evaluation of Frailty and Sarcopenia Markers to Predict Survival in Glioblastoma Patients
Source: J Cachexia Sarcopenia Muscle. 2025 Apr 15;16(2):e13809. doi: 10.1002/jcsm.13809 (PMC11999731; doi:10.1002/jcsm.13809)
Supplement: Supplementary file 10 — Data S1 Supplementary information. [file JCSM-16-e13809-s003.pdf]

STROBE Statement—checklist of items that should be included in reports of observational studies

	Item No.	Recommendation	Page No.	Relevant text from manuscript
**Title and abstract**	1	(*a*) Indicate the study's design with a commonly used term in the title or the abstract	1,2	Title and Abstract
		(*b*) Provide in the abstract an informative and balanced summary of what was done and what was found	2	Abstract
**Introduction**				
Background/rationale	2	Explain the scientific background and rationale for the investigation being reported	3	However, the precision of these markers is limited.
	3	State specific objectives, including any prespecified hypotheses	4	This study aimed to assess the predictive importance of nutritional status in patients with GBM by integrating
**Methods**				
Study design	4	Present key elements of study design early in the paper	5	This study included 309 adult patients with GBM treated at Zhongnan Hospital
	5	Describe the setting, locations, and relevant dates, including periods of recruitment, exposure, follow-up, and data collection	5	Inclusion and exclusion criteria; Data collection
Participants	6	(*a*) *Cohort study*—Give the eligibility criteria, and the sources and methods of selection of participants. Describe methods of follow-up<br>*Case-control study*—Give the eligibility criteria, and the sources and methods of case ascertainment and control selection. Give the rationale for the choice of cases and controls<br>*Cross-sectional study*—Give the eligibility criteria, and the sources and methods of selection of participants	5	Inclusion and exclusion criteria; Data collection

		(*b*) *Cohort study*—For matched studies, give matching criteria and number of exposed and unexposed<br>*Case-control study*—For matched studies, give matching criteria and the number of controls per case	UA	/
Variables	7	Clearly define all outcomes, exposures, predictors, potential confounders, and effect modifiers. Give diagnostic criteria, if applicable	5	Data collection
Data sources/ measurement	8*	For each variable of interest, give sources of data and details of methods of assessment (measurement). Describe comparability of assessment methods if there is more than one group	5	Inclusion and exclusion criteria<br>Data collection
Bias	9	Describe any efforts to address potential sources of bias	5,6	Inclusion and exclusion criteria<br>Statistical analysis
		Explain how the study size was arrived at	5	This study included 309 adult patients with GBM treated at Zhongnan Hospital

Continued on next page

Quantitative variables	11	Explain how quantitative variables were handled in the analyses. If applicable, describe which groupings were chosen and why	6	Statistical analysis
Statistical methods	12	(*a*) Describe all statistical methods, including those used to control for confounding	6	Statistical analysis
		(*b*) Describe any methods used to examine subgroups and interactions	6	Statistical analysis
		(*c*) Explain how missing data were addressed	5,6	Study cohort; Statistical analysis
		(*d*) *Cohort study*—If applicable, explain how loss to follow-up was addressed<br>*Case-control study*—If applicable, explain how matching of cases and controls was addressed<br>*Cross-sectional study*—If applicable, describe analytical methods taking account of sampling strategy	5	Inclusion and exclusion criteria; Data collection
		(*e*) Describe any sensitivity analyses	NA	/
**Results**				
Participants	13*	(a) Report numbers of individuals at each stage of study—eg numbers potentially eligible, examined for eligibility, confirmed eligible, included in the study, completing follow-up, and analysed	7	Clinical characteristics
		(b) Give reasons for non-participation at each stage	7	Clinical characteristics
		(c) Consider use of a flow diagram	/	/
Descriptive data	14*	(a) Give characteristics of study participants (eg demographic, clinical, social) and information on exposures and potential confounders	7	Clinical characteristics
		(b) Indicate number of participants with missing data for each variable of interest	7	Results (Table S1)
		(c) *Cohort study*—Summarise follow-up time (eg, average and total amount)	5	The last follow-up was conducted on 30,December,2023
Outcome data	15*	*Cohort study*—Report numbers of outcome events or summary measures over time	7-8	Results
		Case-control study—Report numbers in each exposure category, or summary measures of exposure	/	/
		Cross-sectional study—Report numbers of outcome events or summary measures	/	/
Main results	16	(*a*) Give unadjusted estimates and, if applicable, confounder-adjusted estimates and their precision (eg, 95% confidence interval). Make clear which confounders were adjusted for and why they were included	7,8	Prognostic Significance of Nutritional Markers in Patients with GBM

	(*b*) Report category boundaries when continuous variables were categorized	7	We determined the optimal threshold for each nutritional indicator using X-tile tools.
	(*c*) If relevant, consider translating estimates of relative risk into absolute risk for a meaningful time period	/	/

Continued on next page

Other analyses	17	Report other analyses done—eg analyses of subgroups and interactions, and sensitivity analyses	7-8	Results
**Discussion**				
Key results	18	Summarise key results with reference to study objectives	9	Our retrospective analysis confirmed that
	19	Discuss limitations of the study, taking into account sources of potential bias or imprecision. Discuss both direction and magnitude of any potential bias	11	Our study has some limitations.
Interpretation	20	Give a cautious overall interpretation of results considering objectives, limitations, multiplicity of analyses, results from similar studies, and other relevant evidence	12	Conclusion
Generalisability	21	Discuss the generalisability (external validity) of the study results	11	Our study has some limitations.
**Other information**				
Funding	22	Give the source of funding and the role of the funders for the present study and, if applicable, for the original study on which the present article is based	13	Funding

*Give information separately for cases and controls in case-control studies and, if applicable, for exposed and unexposed groups in cohort and cross-sectional studies.

Note: An Explanation and Elaboration article discusses each checklist item and gives methodological background and published examples of transparent reporting. The STROBE checklist is best used in conjunction with this article (freely available on the Web sites of PLoS Medicine at http://www.plosmedicine.org/, Annals of Internal Medicine at http://www.annals.org/, and Epidemiology at http://www.epidem.com/). Information on the STROBE Initiative is available at www.strobe-statement.org.
